# Letter from Geo. A. Mills, New York

**Published:** 1890-04

**Authors:** George A. Mills


### CORRESPONDENCE.

NEW YORK, March 10th, 1890.

EDITOR REGISTER :

*Dear Sir:*—We have honored ourselves by honoring our worthy friend and co-laborer, Dr. John Allen, of New York City. This was done by a very finely prepared banquet at the magnificent banqueting rooms of Sperry, in Fifth Avenue. Never was a table for a meeting of dentists so finely laid.

Saturday evening, March 8th, was the appointed time, and when our noble friend, Dr. W. H. Dwinelle, stood at the head of the table and announced to the one hundred and thirty brethren it was a scene that none of those who saw it will ever forget.

Dr. John Allen, fifty-two years in the practice of dentistry, on the left, that Nestor of the dental profession, Dr. W. H. Atkinson, on the right, and in addition there were arranged along the table men of note and prominence, among whom were Drs. Bronson, Clouse, Bonwell, Kingsly, Stockton, Shepherd, Abbott, Jack, Brockway and a host of others.

Dr. E. Parmly Brown was the toast-master for the occasion and we suspect he was the moving spirit that brought together this unusual galaxy of names, and besides all this there were over one hundred letters, all of which contained regret for an enforced absence. Numbers of the letters exhibited a most cordial spirit of brotherhood over the assembled guests; everything passed with all possible smoothness and good will.

Dr. Dwinelle brought all ears to a focus and never did the doctor do himself so much credit; and in reply to the doctor's address Dr. Allen replied in a most genial manner, interspersing reminiscences of rich and rare interest. Following this, were speeches from doctors Kingsly, Jack, Abbott, Shepherd, Clouse, Bonwell, Bartholomew and Mills. This banquet was announced on the card of invitation as a love feast, as in fact it proved.

Do not these things help to prove that "there is nothing mean about us?" that life is worth living? This substantial honor to an exceptionably honorable man is a proof that New York dentists can do something besides gossip, and we are stimulated and strengthened by the fact of having with us and in our midst such men as our noble guest; and we are prompted to a spirit of emulation, and to appreciate, and labor for our calling until it may become second to none.

Fraternally yours, with sincere respect,

George A. Mills.

